# Effects of stemmed and nonstemmed hip replacement on stress distribution of proximal femur and implant

**DOI:** 10.1186/1471-2474-15-312

**Published:** 2014-09-26

**Authors:** Chun-Ming Chen, Wen-Chi Tsai, Shang-Chih Lin, Ching-Shiow Tseng

**Affiliations:** Department of Mechanical Engineering, National Central University, Taoyuan, Taiwan; BoneCare Orthopedic Centers, Han-Chiung Clinics, Taipei, Taiwan; Department of Orthopedic Surgery, National Taiwan University Hospital, Taipei, Taiwan; Graduate Institute of Biomedical Engineering, National Taiwan University of Science and Technology, No. 43, Sec. 4, Keelung Rd, Taipei, 106 Taiwan

**Keywords:** Hip, Stemmed, Nonstemmed, Stress-shielding, Finite-element

## Abstract

**Background:**

Despite improvements in shape, material, and coating for hip stem, both stress shielding and aseptic loosening have been the major drawbacks of stemmed hip arthroplasty. Some nonstemmed systems were developed to avoid rasping off the intramedullary canal and evacuating the bone marrow due to stem insertion.

**Methods:**

In this study, the finite-element models of one intact, one stemmed, and two nonstemmed femora with minimal removal of the healthy neck were investigated to evaluate their biomechanical effects. The resurfacing (ball-shaped) and fitting (neck-shaped) systems were respectively selected as the representative of the ready- and custom-made nonstemmed implants. The stress distribution and interface micromotion were selected as the comparison indices.

**Results:**

The results showed that stress distributions of the two nonstemmed femora are consistently more similar to the intact femur than the stemmed one. Around the proximal femur, the stem definitely induces the stress-shielding phenomenon of its counterparts. The fitting system with the anatomy-shaped cup can make intimate contact with the neck cortex and reduce the bone-cup micromotion and the implant stress. Comparatively, the reamed femoral head provides weaker support to the resurfacing cup causing higher interfacial micromotion.

**Conclusions:**

The reserved femoral neck could act as the load-transferring medium from the acetabular cup, femoral neck, to the diaphysial bone, thus depressing the stress-shielding effect below the neck region. If the hip-cup construct can be definitely stabilized, the nonstemmed design could be an alternative of hip arthroplasty for the younger or the specific patients with the disease limited only to the femoral head.

**Electronic supplementary material:**

The online version of this article (doi:10.1186/1471-2474-15-312) contains supplementary material, which is available to authorized users.

## Background

Total hip replacement (THR) with the intramedullary stem has been successfully employed to treat various hip diseases of the patients over the age of forty [1–3]. The surgical method of the stemmed THR is to amputate the intact femoral neck entirely, rasp off the intramedullary canal, and evacuate the bone marrow for the stem insertion. However, stem insertion into the intramedullary canal necessitates both blood transfusion and bone reaming; and thus might lead to infection [1–5]. If the hip defects are limited to the superficial regions of the femoral head, some studies suggested preserving the bridging bone stock (femoral neck) between the femoral head and the diaphysial shaft [6–9].

There are two types of stem-induced complication that affect the long-term results of THR. One is bone resorption (osteolysis) with the wear debris inducing chemical reaction of the immune system [10]. The other is bone loss (osteoporosis) in response with the stress of the proximal femur shielded by the inserted stem [11–14]. A great many of the clinical results have demonstrated that the stress-shielding effect leads to bone remodeling of the stemmed femur and the implant failure (loosening and cracking) [15–17]. For the diaphysial cortex, the impingement of the stem end has been reported as the stress raiser that induces bone hypertrophy and even fractures [5].

For younger or more active patients, the removal of the more proximal bone stock makes the revision surgery unreliable and even leads to subsequent surgery with increasingly difficult problems and poorer results [1, 5, 15, 17]. In the literatures, some nonstemmed systems have been designed and evaluated by numerical or experimental methods [11, 12, 18–20]. These studies consistently revealed that the proximal region of the nonstemmed femur shows more physiological stress distribution patterns than the stemmed one [11, 12, 18]. The reported biomechanical advantages of the nonstemmed systems over their counterparts were attributed to the preservation of both the femoral neck and the intramedullary canal.

The reported nonstemmed systems can be divided into two types: femoral ball- and neck-shaped cups [18, 21]. Both types preserve the femoral neck and cover the head and neck with the ready- or custom-made cups. The resurfacing system (Durom™, Zimmer, Inc., Indiana, USA) is the typical representative of the ball-shaped cup. Prior to use, the femoral head is reamed as a cylinder to fit the ready-made resurfacing cup. The cancellous cylinder of the reamed ball serves as the load bearer of the superimposed cup. From the biomechanical viewpoint, the femoral neck is subjected to the combined loads, potentially leading to sliding, bending, and twisting of the superimposed cup. Without stem support, hence, the mechanical failure (*e.g.* loosening and breaking) and the construct instability at the bone-implant interfaces have been reported as the major concerns of hip resurfacing system [22, 23].

For the neck-shaped cup, the CT images can be used as the shape reference for designing the custom-made cup that can intimately fit the peripheral cortex of the preserved neck. Without stem insertion, the bone-implant constructs of the two cup systems are stabilized by screws. From the biomechanical viewpoint, the performance of the nonstemmed system is closely related to the load-transferring mechanism of the hip cup, interfacial fitness of the bone-cup construct, and mechanical strength of the underlaid bone (cancellous or cortical). According to the authors’ knowledge, however, there is still no study investigating extensively the biomechanical differences between the ready-made ball- and the custom-made neck-shaped cups.

This study aimed to investigate three subjects of hip arthroplasty: 1) the stem-induced stress-shielding effect on the proximal femur, 2) the biomechanical influence of preserving the femoral neck on the proximal femur, and 3) the micromotion difference between the ball- and neck-shaped cups at the bone-implant interfaces. Based on the CT images, the finite-element model of the intact femur was three-dimensionally reconstructed and the stemmed and nonstemmed hip implants were instrumented. The results of this study would provide insight into the load-transferring and interface-slipping mechanisms of both stemmed and nonstemmed systems.

## Methods

### Femoral model and hip prostheses

**Figure 1:**
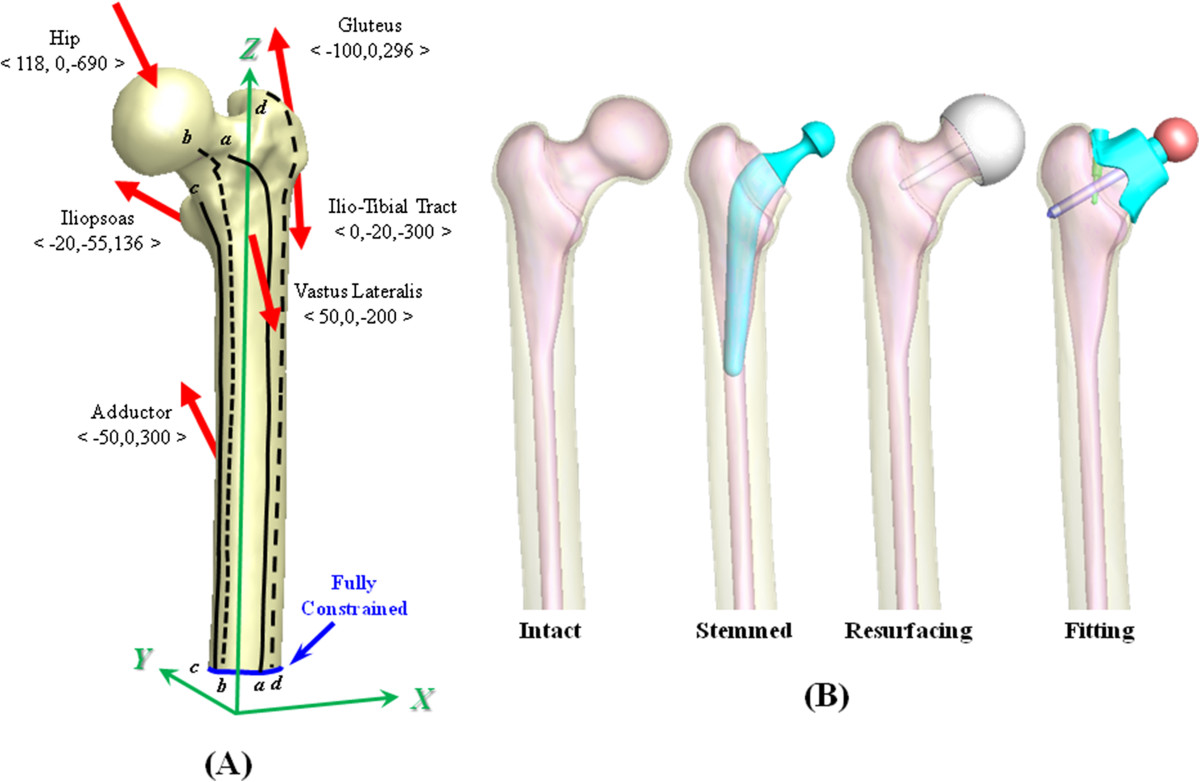
**The finite-element models used in this study. (A)** The model with hip compression and muscular contractions. Four specific lines on the cortical surface were chosen as the comparison indices: anterior line *aa*, posterior line *bb*, medial line *cc*, and lateral line *dd*. **(B)** Four femora were simulated in this study: intact, stemmed, and two nonstemmed.

**Figure 2:**
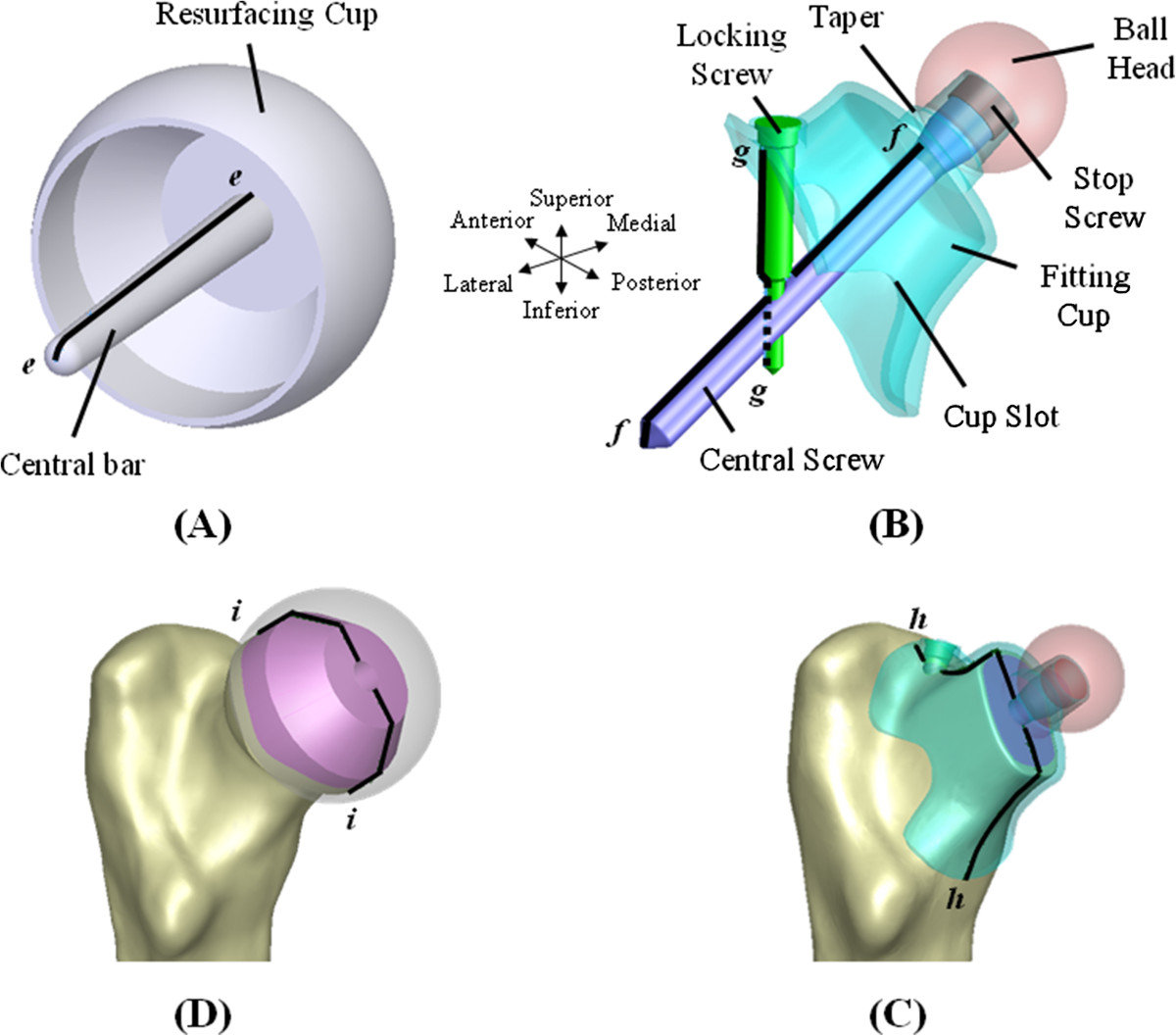
**Three implant and two interface lines were used to evaluate the differences in the distribution of screw stress and bone-cup micromotion. (A)** and **(B)** Implant lines. **(C)** and **(D)** Interface lines.

The pelvis and femur of a 24-year-old male participant without any hip disease were scanned *in vivo* using computed tomography (CT). The participant gave his consent to the collection and utilization of CT-scanning images used in the study. The CT-scanning images with 1-mm slice separation were three-dimensionally reconstructed as a proximal femur with triangular surface meshes using the software PhysiGuide, version 2.3.1 (Pou Yuen Technology Co., Ltd, Changhua, Taiwan) [24]. In this study, the pelvis was excluded and only the femur was used (Figure 1A). The femur consists of the cortical shell and cancellous core whose boundaries were defined from the grayscale difference of the CT image outlines (Figure 1B). The femoral model was further transformed into a solid model with smooth and seamless surfaces using the software SolidWorks, version 2011 (SolidWorks Corporation, Concord, MA, USA). The numerical modeling using CT-scanning images is conformed to the article of “free from ethical approval for scientific research” in the firth term of the article 5 on Research Involving Human Subjects Law by Ministry of Health and Welfare in Taiwan. Therefore, this study is exempt from ethical approval.The intact femur was used as the comparison baseline of the stemmed and nonstemmed systems. The stemmed system was the representative of the conventional hip system (Capital™, 3 M Health Care Ltd., Leicestershire, UK) (Figure 1B). The Durom™ resurfacing cup was the first nonstemmed system comprising a ball head and a central bar (Figures 1B and 2A). For the second nonstemmed system, the current author used the participant’s CT images to design the fitting cup having highly intimate contact with the participant’s neck (Figures 1B and 2B). For such a custom-made cup, a central screw was inserted through the anatomical axis of the femoral neck to further stabilize the fitting cup. If necessary, the use of a locking screw can turn the linkage of two screws into a stability-enhancing mechanism for the bone-cup construct. The cup slot was designed to avoid direct compression on the periarticular blood vessels and nerve roots. For the stemmed model, the bone stock above the neck was obliquely removed and the stem was instrumented into the intramedullary canal, same as in traditional stemmed THR. For the resurfacing and fitting systems, the inner cup makes intimate contact with the underlaid bones. For the fitting system, the threads of the central and locking screws were neglected to simplify the numerical analysis.

### Finite-element analysis

Only the proximal femur was analyzed and the distal end was rigidly fixed (Figure 1A). Both hip compression and muscular contractions were simulated as the physiological loads onto the proximal femur. The *X-Y-Z* coordinate system with the origin at the knee intercondyle was set to define the muscular contractions. The *X*, *Y*, and *Z* axis were mediolaterally, anteroposteriorly, and superoinferiorly directed, respectively (Figure 1A). The insertion points and force directions of the gluteus, piriformis, iliopsoas, ilio-tibial tract, vastus lateralis, and adductor were cited from the literature [25–27]. The vectorial components of the hip and muscle forces on the femur were selected at the static position of one leg stance [18, 28–30]. The geometric models of one stemmed and two nonstemmed systems were developed in the SolidWorks environment (Figure 1).

The stress distribution, interfacial micromotion, and implant failure were the main concerns of this study (Figures 1A and 2). Four lines from *aa* to *dd* were drawn and projected onto the femoral surfaces to evaluate the implant-related effect on the cortical stress (Figure 1A). For the nonstemmed systems, the highly stressed bar and screws make them the potentials for mechanical failure that were evaluated by the stress distribution along the three lines from *ee* to *gg* (Figure 2A and B). The interfacial micromotion was employed to indicate the instability of the bone-cup construct and was determined along lines *hh* and *ii* (Figure 2C and D). The von Mises and maximum principle stresses were respectively used as the comparison indices of the bone and implant stresses.

The Young’s modulus, Poisson’s ratio and bone density were respectively assigned the values of 17.5 GPa, 0.3 and 1.9 gcm^-3^ for the cortical bone and 1.5 GPa, 0.12 and 0.8 gcm^-3^ for the cancellous bone [30–33]. The Co-Cr-Mo alloy with Young’s modulus (=234 GPa) and Poisson’s ratio (=0.3) was used as the material of the stemmed and nonstemmed implants. The friction coefficients were assigned as 0.2 between cup and bone and 0.32 between metallic prostheses, respectively [34, 35]. All materials were assumed to have linearly elastic, homogeneous, and isotropic material properties throughout. The calculated stresses of all implants were compared with the yielding strength (=450 MPa) of the Co-Cr-Mo alloy to validate the assumption of linear elasticity. The interaction of touching surfaces between the bone-implant interfaces were simulated with the surface-to-surface contact elements to allow interfacial slippage.

By using the automatic mesh algorithm, the bone-implant constructs were meshed by the software Simulation, version 2011 (SolidWorks Corporation, Concord, MA, USA). Four models were meshed by the 10-node tetrahedral solid elements with the curved element boundary, thus eliminating the sharp discontinuities to induce unrealistically high stress concentration. According to aspect ratio and Jacobian checks, all elements were within acceptable distortion limits to maximize the accuracy of the results. There was a refinement of the mesh in the vicinity of the aforementioned lines, cups and screws, to improve the modeling accuracy until excellent monotonic convergence behavior (h-adaptive method) with < 1% difference in the total strain energy was achieved. A special element density, which is twice averagely that for the rest of the model, was used for the cups and screws. The overall average element size was 3.5 mm. The numbers of elements of the four models were 92,911 (intact), 74,552 (stemmed), 89,703 (resurfacing), and 100,683 (fitting), respectively.

## Results

### Stress distribution of surrounding bones

**Figure 3:**
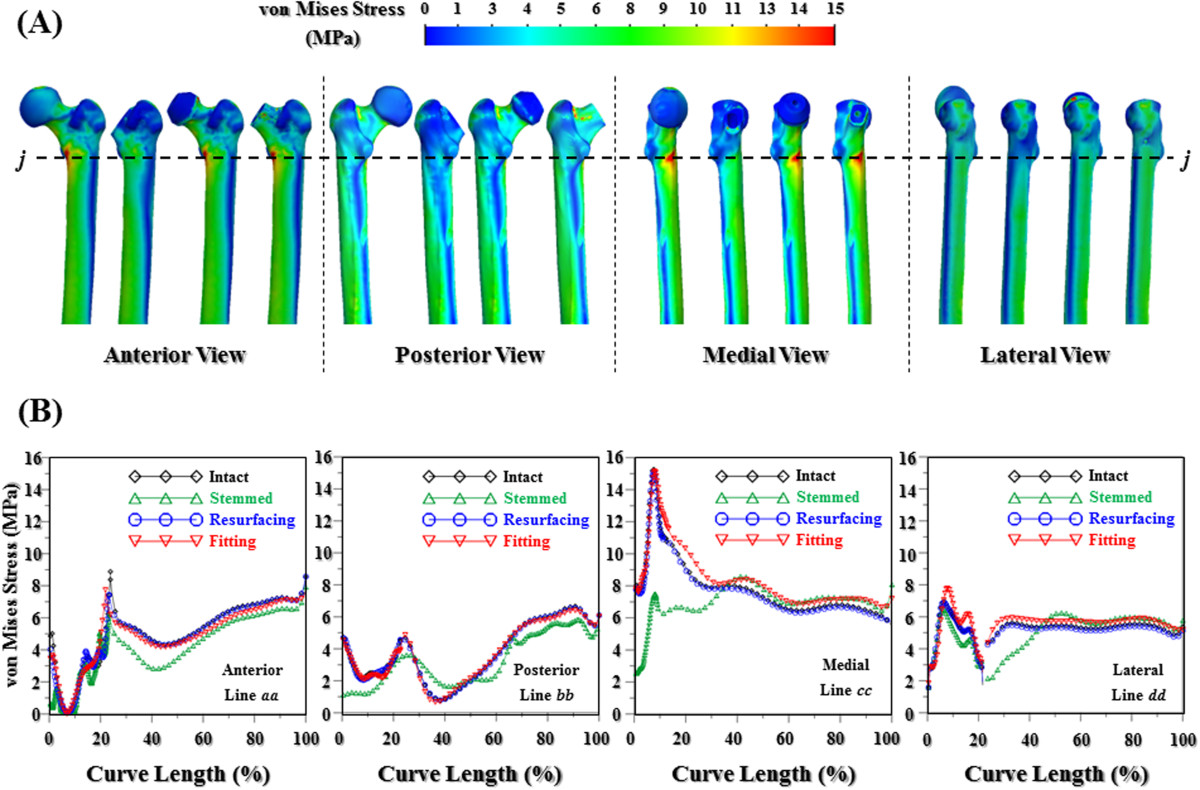
**The distribution patterns of the von Mises stress for the different femoral cortexes. (A)** Stress contours. **(B)** Stress values along the four lines.

The validation steps of this finite-element model had been described in the previous study of the current authors [36]. The von Mises stress distributions of the four femoral cortexes were shown in Figure 3. The stress distribution patterns of the two nonstemmed femora were similar to those of the intact femur at the proximal cortex. For all views, however, the stress values of the stemmed femur were significantly smaller than those of its counterparts. The biomechanical differences between the intact and instrumented femora were quantitatively compared at the line *jj* (Figure 3A). Compared with the intact femur; the stress values of the stemmed femur were averagely decreased about 16.3% (anterior), 16.8% (posterior), 60.6% (medial), and 46.8% (lateral) at the greater trochanter. The aforementioned differences were increased about 5.1% (anterior) and decreased about 2.3% (posterior), 5.5% (medial), and 2.4% (lateral) for the resurfacing system. The differences were increased about 8.0% (anterior), decreased 6.4% (posterior), and increased 8.4% (medial) and 9.5% (lateral) for the fitting system. Among the four femora, the stress difference in the diaphysial cortex was decreased for all sides.

### Micromotion at bone-cup interface

**Figure 4:**
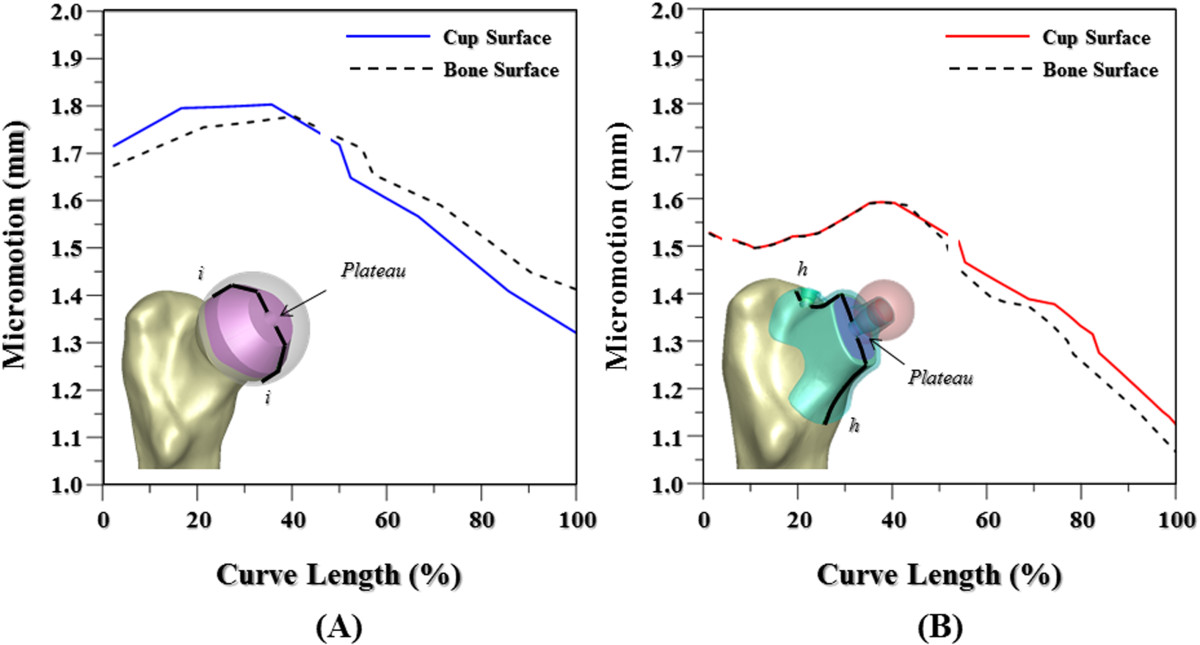
**Interfacial micromotion of the two nonstemmed systems along the two interface lines.** The cup and bone surfaces denoted as the line on the cup interior and bone exterior, respectively. **(A)** Resurfacing system. **(B)** Fitting system.

Upon loading, the interfacial micromotion results in the gap between the resurfacing cup and reamed femur, except for the plateau (Figure 4A). Above the central bar, the micromotion along line *ii* was on average 0.06 mm. Comparatively, the fitting system seems to be more stable in response to the result that the interfacial micromotion is mostly exhibited along the upper line *hh* (Figure 4B). For the two nonstemmed systems, the interfacial micromotion always occurs along the inferior portion of the two interface lines. Below the plateau, the micromotion value of the fitting system was somewhat smaller than that of the resurfacing system.

### Stress distribution of nonstemmed implants

**Figure 5:**
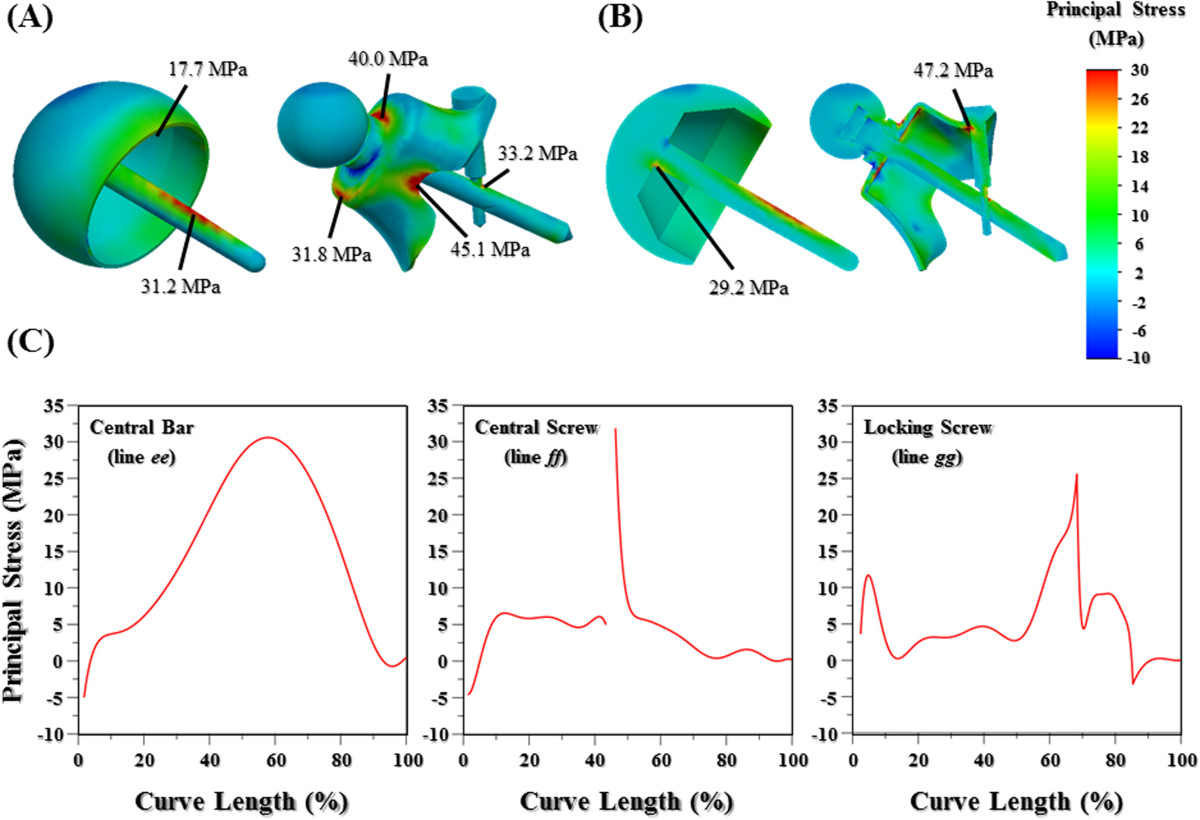
**Contour plots and peak values of the maximum principle stress for the nonstemmed systems. (A)** Isometric view. **(B)** Sectioned view. **(C)** Maximum principle stress along the central bar, central screw, and locking screw.

The maximum principle stress distribution patterns of the nonstemmed systems are shown in Figure 5. For the fitting system, the concave design of the cup slot makes it the stress raiser (45.1 MPa) compared with the other region (Figure 5A). For the resurfacing system, the central bar serves as the stabilizer for the superimposed cup and bears most of the physiological loads, resulting in high stress concentration at the cup-bar junction (29.2 MPa) (Figure 5B). Both hip compression and muscular contraction were transferred to the fitting cup through the central screw and taper, thus leading to stress concentration at the cup-taper junction (40.0 MPa). For the fitting cup, one of the stabilizing mechanisms is the locking screw. Consequently, stress is mostly concentrated at the cup/locking screw junctions (47.2 MPa). The intersecting hole of the central screw also makes it the stress-concentrated site (33.2 MPa). The stress distribution along the central bar was the bell-shaped pattern with the peak value at the middle region (Figure 5C).

## Discussion

### Stress distribution of surrounding bones

From the biomechanical viewpoint, the inserted stem inevitably redistributes the stress values of the proximal femur [11, 13, 14]. Subsequently, the reduced stress and induced debris around the stem make the surrounding bones prone to bone osteoporosis (stress shield) and resorption (immune reaction). These two bone reactions often deteriorate the stem-stabilizing environment. Hence, the weakened bone strength and increased interface micromotion cannot support the external loads through the inserted stem [1, 11–13]. Figure 3 show significant differences in cortical stresses on the medial side between the intact and the stemmed femora. For the nonstemmed femora, however, the cortical stresses within the proximal and diaphysial regions were similar to those of the intact femur.

For the medial cortex below the line *jj* (Figure 3A), the decrease in cortical stress showed that most of the proximal loads were transferred from the stem to the diaphysial cortex [14]. According to Wolff's law, the reduction in bone loads causes the bone to adapt itself by reducing the mechanical strength (material remodeling) or thinning the trabecular size (structural remodeling). The results of the current study were consistent with the reported stem-induced effect on bone stress [11, 13, 14]. This indicates potentially higher aseptic loosening between the intramedullary stem and cancellous bone compared with that of the nonstemmed implant [11, 12, 37], which is consistent with the phenomenon observed for cortical stress. The results of this study demonstrated the stem-induced stress problem on the proximal femur.

### Micromotion at bone-cup interfaces

**Figure 6:**
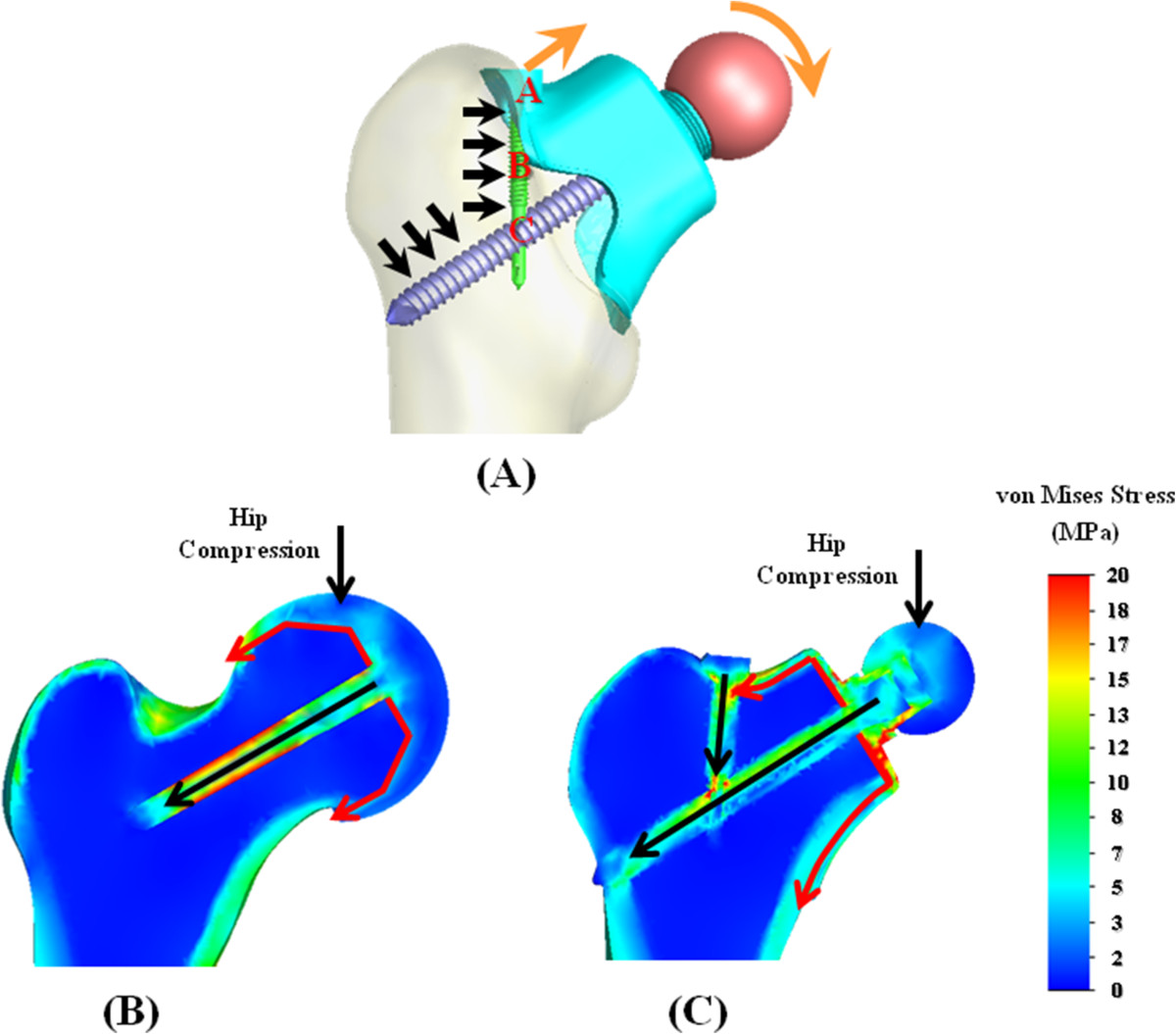
**Schematic diagrams illustrated the load-transferring mechanisms of the nonstemmed systems.** All symbols were described in the content. **(A)** Fitting system. **(B)** and **(C)** Isometric views of the two nonstemmed systems.

Detailed results of the interfacial micromotion for the two nonstemmed systems are shown in Figure 4. For the resurfacing system, significant micromotion along line *ii* exists except for the plateau. The bone-cup micromotion of the resurfacing system is greater than that of the fitting system, especially at the superior region of the interface lines (Figure 4). This can be explained by three structural factors of cup design and bone strength. The first is that the fitting system provides more intimate contact area between the cup interior and the underlaid bone than the resurfacing system (Figure 1B). The second is that the bone-screw interfaces are more stable for the fitting system than for the resurfacing system (Figure 2A and B). The resurfacing cup is stabilized only by the central bar but two intersecting screws are employed to form the interlocking mechanism of the fitting cup. For the fitting system, the loads onto the ball head will medially bend the portion A of the cup, which is immobilized by the locking screw (Figure 6A). However, the pullout of the locking screw will induce clockwise rotation of the screw shaft at portion B and can be reasonably assumed to be constrained by the intersecting mechanism at portion C. The third is that the custom-made cup of the fitting system covers directly the stronger cortex and the resurfacing cup is superimposed over the reamed cancellous bone (Figure 2C and D). Consequently, the differences in contact situation, locking mechanism, and bone strength contribute to greater stability of the fitting than the resurfacing system. As in the resurfacing system, however, the downward movement of the fitting cup inevitably leads to interfacial micromotion at the distal portion of line *hh* (Figure 4B).

### Stress distribution of nonstemmed implants

Contrary to the stemmed system, the ball-shaped cup is supported by the underlaid bone and further stabilized by the central bar. However, this makes the central bar as the heavily stressed component (Figure 5). Except for the cup-bar junction, stress concentration also occurs at approximately the middle region of the central bar where is about projective site of cup rim onto the bar (Figure 5A and B). It can be explained by the fact that, outside the cup, the bar begins to bear the loads. For the fitting system, the intercomponent junctions and cup slots were the stress-concentrated sites. Owing to shape discontinuity, the highly stressed sites of the central and locking screws are the interlocking hole and the diameter-reduced corner (Figure 5C). The designs should be modified to avoid plastic yielding or fatigue cracking at these sites. Results of this study showed that the long-term stabilization of the bone-cup construct is the major concern of the nonstemmed system without stem insertion.The load-transferring mechanisms of the two nonstemmed systems are compared in Figure 6B and C. Hip compression is the dominant load at the femoral head while anteroposterior loads are comparatively minor, resulting in comparable values of cortical stress between the resurfacing and fitting systems (Figure 3B). For the resurfacing system, hip compression was transferred into the proximal femur through the cup interior (red arrows) and central bar (black arrow) (Figure 6B). For the fitting system, the load-transferring paths are the cup interior (red arrows) and the central screw (black arrow) (Figure 6C). The load through the upper contact surface of the fitting cup is further transferred through the locking screw. The load through the central screw is concentrated at the intersecting site and transferred into the lateral cortex. Due to support of lateral cortex and longer bone-screw contact, the peak stress at the intersecting site of the central screw was somewhat higher than that of the central bar (Figure 5A).

## Conclusions

The stem insertion induces stress redistribution of the proximal and diaphysial femur. Less removal of the proximal bone stock makes the load-transferring pattern of the nonstemmed femur more physiologically similar to that of the intact femur. Without the inserted stem, the central bar of the resurfacing system was highly stressed, making it prone to mechanical failure. Both contact situation and bone strength should be determinant factors of long-term stability of the nonstemmed system. For the cup-shaped system, the cup should be the custom-made and the underlaid cortex could be preserved to provide more intimate and stronger support to the superimposed cup. If the cup can be definitely stabilized, the nonstemmed design could be an alternative for the younger or the specific patients with the disease limited only to the femoral head.

## Authors’ original submitted files for images

Below are the links to the authors’ original submitted files for images. Authors’ original file for figure 1 Authors’ original file for figure 2 Authors’ original file for figure 3 Authors’ original file for figure 4 Authors’ original file for figure 5 Authors’ original file for figure 6

## Abbreviations

THR: Total hip replacement
CT: Computed tomography.
